# Supplementary material for: Content-rich biological network constructed by mining PubMed abstracts
Source: BMC Bioinformatics. 2004 Oct 8;5:147. doi: 10.1186/1471-2105-5-147 (PMC528731; doi:10.1186/1471-2105-5-147)
Supplement: Additional File 5 — The original Chilibot query results of the term "long-term potentiation (LTP)" and 22 other terms, limiting the latest references analyzed to the years 1990, 1995, 2000, and 2004. [file 1471-2105-5-147-S5.bz2 › chilibotAdditionalFile5/ltp2000/html/TRKB_ERK.html]

 


 **TRKB** and **ERK** 
  
Found 9 abstracts in PubMed,  **9 abstracts were retrieved and analyzed**.  


---

 Search Google  |
 PDF files only 
|  EDU domain only 

---

- J Neurochem, 2000   Brain derived neurotrophic factor is known to have important functions in neuronal survival, differentiation, and plasticity.
  In addition to its role as a survival promoting factor, BDNF reportedly can enhance neuronal cell death in some cases, for example, the death caused by excitotoxicity or glucose deprivation.
  The cellular mechanism of the death enhancing effect of BDNF remains unknown, in contrast to that of its survival promoting effect.
  In this work, we found that BDNF markedly accelerated the nitric oxide donor induced death of cultured embryonic cortical neurons.
  BDNF increased the number of cells with nuclear condensation and DNA fragmentation 24 h after treatment with the NO donor, but it did not change the number of those cells 36 h after the treatment.
  The BDNF accelerated death of cortical neurons was inhibited by the addition of actinomycin D or cycloheximide.
  These results suggest that BDNF can accelerate apoptotic cell death elicited by NO donor.
  **TrkB** IgG and K252a blocked the BDNF induced acceleration of the death, indicating that the death accelerating effect by BDNF is mediated by **TrkB**.
  In addition, the BDNF accelerated apoptosis was inhibited by the addition of SB202190 and SB203580, specific inhibitors of p38 mitogen activated protein kinase , and U0126, a specific inhibitor of MAPK **ERK** kinase 1, indicating that the activation of both p38 MAPK and **ERK** is involved in the signaling cascade of the BDNF accelerated, NO donor induced apoptosis.

  - Invest Ophthalmol Vis Sci, 2000   PURPOSE To investigate neurotrophic growth factors and corresponding receptors in human and rabbit corneal epithelium and stroma.
    METHODS Transcription of nerve growth factor , neurotrophin 3 , NT 4, brain derived neurotrophic factor , glial cell line derived neurotrophic factor , and receptors Trk A E, was investigated by reverse transcription polymerase chain reaction.
    DNA dot blot analysis allowed to estimate transcription levels.
    Single cell proliferation assays were performed using recombinant NGF, BDNF, and GDNF.
    Mitogen activated protein kinase signal transduction was investigated with Western blot analysis using antibodies against activated and total extracellular signal regulated kinase 1 2 and the jun N terminal protein kinase 1 2.
    RESULTS Transcription of NGF, NT 3, BDNF, and Trk A, Trk B **[TRKB]**, Trk C, and Trk E receptors was detected in both ex vivo and cultured epithelium and stroma.
    Transcription of NT 4 was only detected in epithelium and transcription of GDNF only in stroma.
    Levels of transcription were higher for NT 3, NT 4, and the Trk receptors and lower for NGF, BDNF, and GDNF.
    NGF and GDNF stimulated both epithelial colony formation and proliferation.
    . . . BDNF only enhanced colony formation.
    Stromal proliferation was enhanced in serum free medium.
    In epithelium, predominantly **ERK** 1 was activated by NGF, GDNF, and BDNF.
    In stromal cells NGF and GDNF stimulated phosphorylation of **ERK** 1 and JNK 1.
    CONCLUSIONS Neurotrophic factors and tyrosine kinase receptors are transcribed in the human cornea.
    GDNF and NGF stimulate corneal epithelial proliferation, and the effect of the latter might be mediated by activation of **ERK** 1.
    Neurotrophic factors have very specific effects on phosphorylation of **ERK** and JNK in epithelial and stromal cells.
    The differential expression of NT 4 and GDNF suggests a regulatory function within the cytokine network of the cornea.

    - J Cell Sci, 2000   Phosphorylation of the neurofilament H subunit was investigated in rat embryonic brain neurons in culture.
      A portion of the NF H was phosphorylated in vivo at embryonic day 17 when brain neurons were prepared.
      When the neurons were isolated and cultured, the NF proteins disappeared once and then reappeared over the next several days in the following order NF L NF M, dephosphorylated NF H and phosphorylated NF H.
      Phosphorylation of NF H began around 4 days after cell plating, at about the time of synapse formation.
      Treatments that appeared to modulate the timing of synapse formation also affected the timing of NF H phosphorylation earlier phosphorylation was observed at higher neuronal cell density, earlier phosphorylation was observed in neurons cultured on a coating substrate that promotes rapid neurite extension and phosphorylation was suppressed when neurite extension was inhibited by brefeldin A.
      Three possible synapse formation induced events, excitation, cell cell contact through adhesion proteins and elevated concentrations of neurotrophic factors, were examined for their possible involvement in generating the signal for NF H phosphorylation.
      Neither excitation nor cell contact enhanced NF H phosphorylation.
      Neurotrophic factors, brain derived neurotrophic factor and neurotrophin 3 stimulated phosphorylation of NF H.
      The BDNF stimulated phosphorylation was inhibited by an anti BDNF antibody and K252a, an inhibitor of BDNF receptor **TrkB** tyrosine kinase.
      Among known NF H kinases of cyclin dependent kinase 5 , external signal regulated protein kinase and stress activated protein kinase , CDK5 and SAPK showed an increase in kinase activity or an active form with a time course similar to NF H phosphorylation in control culture.
      On the other hand, BDNF stimulated the kinase activity of CDK5 and induced appearance of an active form of **ERK** transiently.
      These results suggest a possibility that synapse formation induces NF H phosphorylation, at least in part, through activation of CDK5 by BDNF.

      - J Recept Signal Transduct Res, 1999   The **TrkB** receptor tyrosine kinase regulates cellular proliferation via signal transduction pathways involving SHC, PLCgamma, and CBL.The **TrkB** protein tyrosine kinase is a high affinity receptor for brain derived neurotrophic factor and neurotrophin 4 .
        **TrkB** autophosphorylation occurs on five cytoplasmic tyrosines Y484, Y670, Y674, Y675, and Y785.
        Using site directed mutagenesis, we have assessed the importance of **TrkB** tyrosines 484 and 785 in affecting **TrkB** mediated signaling events leading to NIH 3T3 cell mitogenesis and survival.
        Mutation of **TrkB** tyrosine 484, while having no affect on BDNF inducible PLCgamma and Cbl tyrosine phosphorylation, is essential for the phosphorylation of Shc, the complete activation of extracellular regulated kinase 1 2 and the induction of c fos protein synthesis.
        In contrast, mutation of Y785 does not significantly affect BDNF inducible Shc phosphorylation, ERK1 2 activation, or c fos protein synthesis, but completely inhibits the tyrosine phosphorylation of PLCgamma and Cbl.
        These data indicate that both **ERK** dependent and **ERK** independent signaling pathways lead to BDNF inducible mitogenesis and survival.

        - J Neurochem, 1999   **Extracellular regulated kinases and phosphatidylinositol 3 kinase are involved in brain derived neurotrophic factor mediated survival and neuritogenesis of the neuroblastoma cell line SH SY5Y.** .
          Retinoic acid RA induces the differentiation of many cell lines, including those derived from neuroblastoma.
          RA treatment of SH SY5Y cells induces the appearance of functional Trk B **[TRKB]** and Trk C receptors.
          Acute stimulation of RA predifferentiated SH SY5Y cells with brain derived neurotrophic factor BDNF , neurotrophin 3 NT 3 , or neurotrophin 4 5 NT 4 5 , but not nerve growth factor NGF , induces Trk autophosphorylation, followed by phosphorylation of Akt and the extracellular signal regulated kinases ERKs 1 and 2.
          In addition, BDNF, NT 3, or NT 4 5, but not NGF, promotes cell survival and neurite outgrowth in serum free medium.
          The mitogen activated protein kinase and **ERK** kinase MEK inhibitor PD98059 blocks BDNF induced neurite outgrowth and growth associated protein 43 expression but has no effects on cell survival.
          On the other hand, the phosphatidylinositol 3 kinase inhibitor LY249002 reverses the survival response elicited by BDNF, leading to a cell death with morphological features of apoptosis.

          - Exp Neurol, 1999   **Early BDNF, NT 3, and NT 4 signaling events.** .
            Much more is known about nerve growth factor NGF signaling than that initiated by brain derived neurotrophic factor BDNF , neurotrophin 3 NT 3 , or NT 4.
            We sought to study early BDNF, NT 3, and NT 4 signaling events.
            Using **TrkB** expressing cells, we found that BDNF and NT 4 individually induced tyrosine phosphorylation of **TrkB** in a dose dependent fashion.
            At maximally effective concentrations, BDNF or NT 4 induced robust **TrkB** tyrosine phosphorylation at 5 min.
            this progressively declined at 1 0, and 60 min.
            Using immunoprecipitation, PI3 kinase and tyrosine phosphorylated PLC gamma1 and SHC were shown to be associated with tyrosine phosphorylated **TrkB** in response to both BDNF and NT 4.
            BDNF and NT 4 induced similar intensities of phosphorylation of **TrkB** and signaling intermediates at equivalent doses.
            NT 3 treatment of TrkC expressing cells induced very similar patterns for induction of TrkC tyrosine phosphorylation and recruitment of signaling intermediates.
            BDNF, NT 3, and NT 4 caused rapid tyrosine phosphorylation of **ERK** and SNT.
            These data suggest that the earliest signaling events for BDNF, NT 3, and NT 4 are very similar to those for NGF.

            - J Neurochem, 1998   Monoamine activated alpha2 macroglobulin was shown to reduce the dopamine concentration in corpus striatum of adult rat brains and inhibit other neuronal functions in vivo and in vitro.
              As brain derived neurotrophic factor, neurotrophin 4, and neurotrophin 3 are important neurotrophic factors for dopaminergic neurons, the effect of monoamine activated alpha2M on signal transduction by **trkB** and trkC was investigated.
              The results show that monoamine activated alpha2M binds to **trkB** and inhibits brain derived neurotrophic factor neurotrophin 4 promoted autophosphorylation of **trkB** in a dose dependent manner in both **trkB** expressing NIH3T3 and human neuroblastoma SH SY5Y cells.
              Monoamine activated alpha2M also blocks tyrosine phosphorylation of phospholipase C gamma1 and extracellular signal regulated protein kinase 1, which are key intracellular proteins involved in **trkB** signal transduction.
              Similarly, monoamine activated alpha2M inhibits tyrosine phosphorylation of neurotrophin 3 induced trkC and its signal transduction in a dose dependent manner in NIH3T3 cells expressing trkC .
              In contrast to monoamine activated alpha2M, normal alpha2M has little or no significant inhibitory effect on the phosphorylation of **trkB** and trkC.
              In addition, the retinoic acid promoted tyrosine phosphorylation of phospholipase C gamma1, **ERK** 1, and or **ERK** 2 in SH SY5Y cells was unaffected by monoamine activated alpha2M.
              this suggests that the inhibitory effect of activated alpha2M on the neurotrophin stimulated phosphorylation of intracellular signalling proteins may be specific.
              Taken together, the data indicate that activated alpha2M is a pan trk inhibitor, which by virtue of its binding to trk receptors may block trk mediated signal transduction in dopaminergic neurons and lead to reduction of dopamine concentration in corpus striatum.

              - Blood, 1997   Expression of functional TrkA receptor tyrosine kinase in the HMC 1 human mast cell line and in human mast cells.Nerve growth factor can influence mast cell development and function in murine rodents by interacting with its receptors on mast cells.
                We now report the identification of mRNA transcripts of full length tyrosine kinase containing trkA, **trkB**, and trkC neurotrophin receptor genes in HMC 1 human mast cell leukemia cells.
                Although HMC 1 cells lacked p75 mRNA, they expressed transcripts for the exon lacking splice variant of trkA , truncated **trkB** , and truncated trkC.
                By flow cytometry, HMC 1 cells exhibited expression of TrkA, **TrkB**, and TrkC receptor proteins containing full length tyrosine kinase domains.
                NGF stimulation of HMC 1 cells induced tyrosine phosphorylation of TrkA protein, increased expression of the early response genes c fos and NGF1 A, and activation of **ERK** mitogen activated protein kinase, results which indicate that TrkA receptors in HMC 1 cells are fully functional.
                Highly purified populations of human lung mast cells expressed mRNAs for trkA, **trkB** and trkC.
                . . . preparations of human umbilical cord blood derived mast cells expressed mRNAs for trkA and trkC, but not **trkB**.
                Moreover, preparations of human umbilical cord blood derived immature mast cells not only expressed mRNA transcript and protein for TrkA, but exhibited significantly higher numbers of chymase positive cells after the addition of NGF to their culture medium for 3 weeks.
                In addition, HMC 1 cells expressed mRNAs for NGF, brain derived neurotrophic factor , and neurotrophin 3 , the cognate ligands for TrkA, **TrkB**, and TrkC.
                . . . NGF and BDNF transcripts were detectable in human umbilical cord blood mast cell preparations.
                Taken together, our findings show that human mast cells express a functional TrkA receptor tyrosine kinase and indicate that NGF may be able to promote certain aspects of mast cell development and or maturation in humans.
                Our studies also raise the possibility that human mast cells may represent a potential source for neurotrophins.
